# Retrospective multicentric survival analysis of patients receiving TPEx regimen as first-line treatment of recurrent and/or metastatic head and neck squamous cell carcinoma

**DOI:** 10.1016/j.esmoop.2025.104544

**Published:** 2025-04-11

**Authors:** L. Libert, C. Abdeddaim, K. Saleh, C. Even, S. Duplomb, J. Dubreuil, A. Rambeau, E. Guiard, Y. Pointreau, N. Olympios-Gerotzortzos, C. Moldovan, E. Lévêque, F. Clatot

**Affiliations:** 1 Department of Medical Oncology, Centre Henri Becquerel, Rouen, France; 2 Faculté de Médecine et Pharmacie de Rouen, University of Rouen, Rouen, France; 3 Department of Medical Oncology, Centre Oscar Lambret, Lille, France; 4 Department of Medical Oncology, Institut Gustave Roussy, Villejuif, France; 5 Department of Medical Oncology, Hospices Civils de Lyon, Lyon, France; 6 Department of Medical Oncology, Centre François Baclesse, Caen, France; 7 Department of Radiotherapy, Institut inter-régionaL de Cancérologie (ILC) – Centre Jean Bernard, Centre de Cancérologie de la Sarthe, Le Mans, France; 8 Clinical Research Unit, Centre Henri Becquerel, Rouen, France

**Keywords:** squamous cell carcinoma of head and neck, metastatic, antineoplastic combined chemotherapy protocols, platinum, cetuximab, immunotherapy, PD-1 inhibitors

## Abstract

**Background:**

TPEx regimen (docetaxel, platinum, cetuximab) is a first-line treatment option for recurrent and/or metastatic head and neck squamous cell carcinoma (R/M HNSCC) if combined positive score <1, or in case of high tumor burden. We sought to evaluate the survival rates of patients receiving TPEx in real life as first-line treatment of R/M HNSCC, particularly since the advent of immunotherapy (IO) as second-line therapy.

**Methods:**

This multicentric retrospective study included patients treated by a first cycle of TPEx between 2018 and 2023, with a performance status of 0 or 1. The primary endpoint was overall survival (OS). Secondary end-points were progression free survival (PFS1), rate of patients exposed to IO after TPEx, PFS of patients on second line treatment (PFS2).

**Results:**

A total of 204 patients were included, mainly men (86%), previously treated for a localized HNSCC (78%). Some 32% of patients had a clinically threatening disease. Combined positive score was available for 88 patients (43%). Patients were treated with a median of four cycles of TPEx, followed by cetuximab maintenance for 154 patients. After a median follow-up of 35.8 months, median OS was 17.9 months [95% confidence interval (CI) 15.7-19.6 months], median PFS1 was 6.0 months (95% CI 5.7-6.9 months) and median PFS2 was 2.5 months (95% CI 2.0-2.8 months). Among the 182 patients who progressed under TPEx, 148 patients were exposed to IO (81.3%) in subsequent lines.

**Conclusion:**

Median OS of 17.9 months under TPEx as first-line treatment of R/M HNSCC compares favorably with historical data. IO exposure after progression on TPEx was the rule.

## Highlights

• Real life TPEx in PS 0-1 patients led to a median OS of 17.9 months.
• More than 80% of patients were exposed to immunotherapy after progression on TPEx.
• Patients with CPS ≥1 frequently had a clinically threatening disease.
• Half of the patients had at least three lines of treatment in the metastatic setting.

## Introduction

Head and neck cancer is the eighth most common cancer worldwide with an annual incidence of 890 000 cases, most of them being squamous cell carcinomas.^1^^,^^2^ Tobacco, alcohol and human papilloma virus are known risk factors.^3^^,^^4^ If only 10% of patients are diagnosed with a *de novo* metastatic stage, 50% of patients with locally advanced disease (T3-T4 and/or N+) will relapse to a locoregional and/or metastatic stage within 3 years of treatment completion.^5^^,^^6^

In Europe, first-line treatment of patients with recurrent and/or metastatic head and neck squamous cell carcinoma (R/M HNSCC) ineligible to curative treatment is guided by programmed death-ligand 1 (PD-L1) tumor expression,^7^ assessed by the combined positive score (CPS), defined as the number of PD-L1-staining cells (tumor cells, lymphocytes and macrophages) divided by the total number of tumor cells multiplied by 100.^8^

According to European Medicines Agency (EMA) approval, if CPS ≥1, IO (immunotherapy) alone or chemo-immunotherapy (platinum and 5-fluorouracil-based protocol) are available.^7^ If CPS <1, the standard of care (SoC) is a platinum-based chemotherapy combined with cetuximab, an epidermal growth factor receptor (EGFR) antibody.^7^ These guidelines are based on the results of *Keynote048*^9^ in which the EXTREME arm (5-fluorouracil–platinum–cetuximab) had a lower overall survival (OS) compared with the pembrolizumab alone arm for CPS ≥1 population and compared with the chemo-immunotherapy arm (5-fluorouracil–platinum–pembrolizumab) in the CPS ≥1 and the total populations. A subgroup analysis, however, showed no significant difference in OS between EXTREME and chemo-immunotherapy arms in CPS <1 subgroup.^10^

In the TPExtreme^11^ randomized phase II trial, the TPEx protocol (docetaxel–platinum–cetuximab) was compared with the EXTREME protocol in first-line setting for R/M HNSCC. While there was no significant difference in OS between the two arms (13.4 months in the EXTREME arm versus 14.5 months in the TPEx arm), the TPEx group showed fewer toxicities, fewer dose adjustments and better quality of life. Moreover, the very low rate of early progression under TPEx (9%) makes this protocol interesting in case of threatening disease. Thus, TPEx became an alternative SoC for R/M HNSCC for CPS <1 tumors.^7^

Even if no direct comparison can be made, the OS of patients in the EXTREME arm in Keynote048^9^ (10.7 months) appears to be shorter than in more recent studies (13.5 months in CheckMate651^12^ and 13.4 months in TPExtreme^11^). One possible explanation is the low exposure rate (25%) to IO after EXTREME in Keynote048.^9^ Nevertheless, EMA approved nivolumab in second line since 2017 with the results of Checkmate141^13^ demonstrating superiority of nivolumab (anti-PD-1, programmed cell death protein 1) over SoC after platinum-based chemotherapy, regardless of PD-L1 status.

The first-line treatment decision for R/M HNSCC remains heterogeneous,^14^ and TPEx is still used, even for CPS ≥1 tumors in case of high tumor burden or contraindication to 5-fluorouracil.^15^ Since the advent of IO as second-line therapy, however, the survival rates of patients treated by TPEx in first-line setting, all PD-L1 status combined, are unknown. The percentage of patients who will never be exposed to IO after TPEx is also unknown.

The primary aim of this study was to evaluate the survival rates of patients receiving TPEx as first-line treatment of R/M HNSCC. We also wanted to determine the rate of IO exposure after TPEx.

## Material and methods

### Study design and participants

This was a retrospective, observational and multicentric study carried out in six French centers. Eligible patients had histologically confirmed diagnosis of R/M HNSCC not suitable for curative treatment; were ≥18 years; did not object to the use of personal medical data for cancer research; had a performance status (PS) score of 0 or 1; received TPEx regimen as first-line treatment in the R/M setting with at least the cycle 1 day 1 (C1D1) at full dose (docetaxel 75 mg/m^2^ every 3 weeks; cisplatin 75 mg/m^2^ or carboplatin AUC 5 with a minimum of 400 mg every 3 weeks; cetuximab 400 mg/m^2^ for induction dose then 250 mg/m^2^ weekly) between 1 January 2018 and 30 June 2023. Patients who had doses modifications after day 1 for toxicity management were not excluded. We did not include patients treated by TPEx before 2018 as there was no IO available in daily practice in France before that date.

Even though the use of granulocyte-colony stimulating factor was not specifically recorded for the patients included, it is systematically prescribed as part of the TPEx protocol in routine practice.

Exclusion criteria were: locally advanced disease with potential curative treatment; severe allergic reaction to cetuximab at first exposure resulting in its definitive interruption; previous exposure to IO for a head and neck cancer; previous exposure to IO for any other cancer subtypes in the past 3 years; history of other malignancies with active treatment in the previous year; synchronous primary cancer at R/M HNSCC diagnosis.

Data were registered retrospectively using the patient’s electronic file. Baseline characteristics were collected including age, sex, history of treatment of a localized HNSCC, CPS if available, date of diagnosis and if it was a non-operable/non-irradiable recurrent disease or a metastatic disease. Cellulitis and permeation nodules in the head and neck zone were considered metastatic stage. Whether the disease was considered clinically threatening (hyperalgesia requiring use of opioid, active bleeding, extensive laryngeal involvement, extensive pulmonary involvement or lymphangitis, invasion of a main vascular axis, cranial base involvement, cellulitis or permeation nodules) and whether liver metastases were present at diagnosis were assessed.

Data regarding treatments were also collected, such as number of cycles of TPEx, duration of maintenance by cetuximab, exposure to curative-intent closure radiotherapy after TPEx in case of an excellent response, date of progression under TPEx regimen if it occurred, second-line systemic treatment, exposure to anti-PD-1/PD-L1 IO for R/M disease management, date of death or date of last follow-up.

This study was made in compliance with the French Reference Methodology MR-004 and was approved by the institutional review board of Henri Becquerel Center (n° IRB = 2304B).

### Endpoints

The primary endpoint was OS, defined as time from start of treatment by TPEx to death from any cause.

Secondary endpoints were: progression-free survival (PFS) under TPEx (PFS1), defined as time from start of TPEx to first disease progression or death; rate of patients receiving a systemic second-line treatment; rate of patients exposed to IO; PFS of patients on second-line treatment (PFS2), defined as time from start of systemic second-line treatment to progression or death; PFS of patients on second-line IO (PFS2^IO^), defined as time from start of IO (nivolumab or in trials) to progression or death.

OS, PFS1 and PFS2^IO^ were also assessed according to CPS (<1 or ≥1) when available.

Progression could be based on clinical, radiological or anatomopathological examination. Diagnosis of a new primary head and neck tumor was considered as progression.

### Statistical analysis

Qualitative variables were described using headcount and percentage. Quantitative variables were described by mean and standard deviation, median and interquartile range, and extreme values.

OS and PFS were estimated using the nonparametric Kaplan–Meier estimator. Survival delays were calculated from the first day of TPEx protocol. A log-rank test was used to estimate the differences between the survival curves according to the different modalities of the potential prognostic factors. Univariate Cox models were estimated and the results are presented with the associated hazard ratios [95% confidence interval (CI)]. Multivariate models were defined with significant univariate variables. Median follow-up was calculated from the reverse Kaplan–Meier method.

## Results

### Baseline characteristics

Overall, 204 patients were included, 159 of whom (78%) were previously treated for a localized HNSCC. At inclusion, 31.9% of patients had clinically threatening disease and 7.4% had liver metastases. CPS was available for 88 patients (43%), 37.5% of whom had a CPS ≥1. Main characteristics are detailed in Table 1.

**Table 1:** Patients characteristics

Total population	*n* = 204
Sex, *n* (%)	
Male	176 (86.3)
Female	28 (13.7)
Age, years	
Median (IQR)	62 (55-65)
Clinically threatening disease, *n* (%)	
No	139 (68.1)
Yes	65 (31.9)
Liver metastases, *n* (%)	
No	189 (92.6)
Yes	15 (7.4)
History of localized HNSCC, *n* (%)	
No (*de novo* metastatic)	45 (22.1)
Yes (recurrent), time to relapse ≤6 months, *n* (%)	45 (22.1)
Yes (recurrent), time to relapse >6 months, *n* (%)	114 (55.9)
Treatment (*n* = 159)	
Surgery alone	9 (5.7)
Radiotherapy alone	12 (7.5)
Chemoradiotherapy	46 (28.9)
Surgery + radiotherapy	42 (26.4)
Surgery + chemoradiotherapy	49 (30.8)
Other	1 (0.6)
Platinum exposure (*n* = 159)	
No	54 (34.0)
Yes	105 (66.0)
Site of relapse (*n* = 159)	
Locoregional alone	74 (46.5)
Metastatic (± locoregional)	85 (53.5)
CPS availability, *n* (%)	
No	116 (56.9)
Yes	88 (43.1)
Value (*n* = 88)	
CPS <1	55 (62.5)
1≤ CPS <20	25 (28.4)
CPS ≥20	8 (9.1)
Disease stage at CPS analysis^a^ (*n* = 88), *n* (%)	
Localized stage	22 (25.0)
R/M stage	64 (72.7)
Unknown	2 (2.3)

CPS, combined positive score; HNSCC, head and neck squamous cell carcinoma; IQR, interquartile range; R/M, recurrent and/or metastatic.

a Stage at which tumor tissue was sampled for CPS analysis; it could be analyzed on the primary tumor site or on a metastatic site.

### TPEx regimen

Patients were treated with a median of four cycles of TPEx, mainly with cisplatin at C1D1 (61.8%) (Table 2). Cetuximab maintenance was initiated in 154 patients (75.5%), with a mean duration of 5.3 months. After TPEx treatment, 21 patients (10.3%) received a curative-intent closure radiotherapy of the primary tumor and eventually of metastases.

**Table 2:** TPEx regimen

Total population	*n* = 204
Choice of platinum at C1D1, *n* (%)	
Cisplatin	126 (61.8)
Carboplatin	78 (38.2)
Number of TPEx cycles	
Median (IQR)	4.0 (4.0-4.0)
Mean (± SD)	3.7 (±0.9)
Cetuximab maintenance, *n* (%)	
No	50 (24.5)
Yes	154 (75.5)
Duration of maintenance^a^ (months, *n* =144)	
Median (IQR)	3.0 (2.0-6.0)
Mean (± SD)	5.3 (± 6.8)
Curative-intent closure radiotherapy, *n* (%)	
No	183 (89.7)
Yes	21 (10.3)
Dose (Gray, *n* = 21)	
Median (IQR)	70.0 (70.0-70.0)
Mean (± SD)	67.1 (± 7.4)

C1D1, cycle one day one; IQR, interquartile range; R/M, recurrent and/or metastatic; SD, standard deviation; TPEx, docetaxel, platinum, cetuximab.

a For the duration of maintenance, data were missing for 10 patients still being under cetuximab at end of data collection.

### Primary and secondary endpoint results

**Figure 1:**
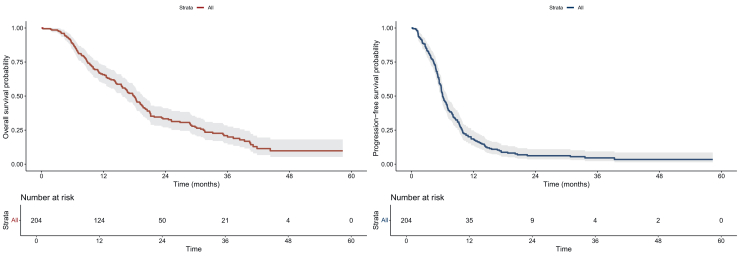
**Kaplan–Meier analysis of OS and PFS1 in total population.** CI, confidence interval; OS, overall survival; PFS1, progression-free survival 1.

**Figure 2:**
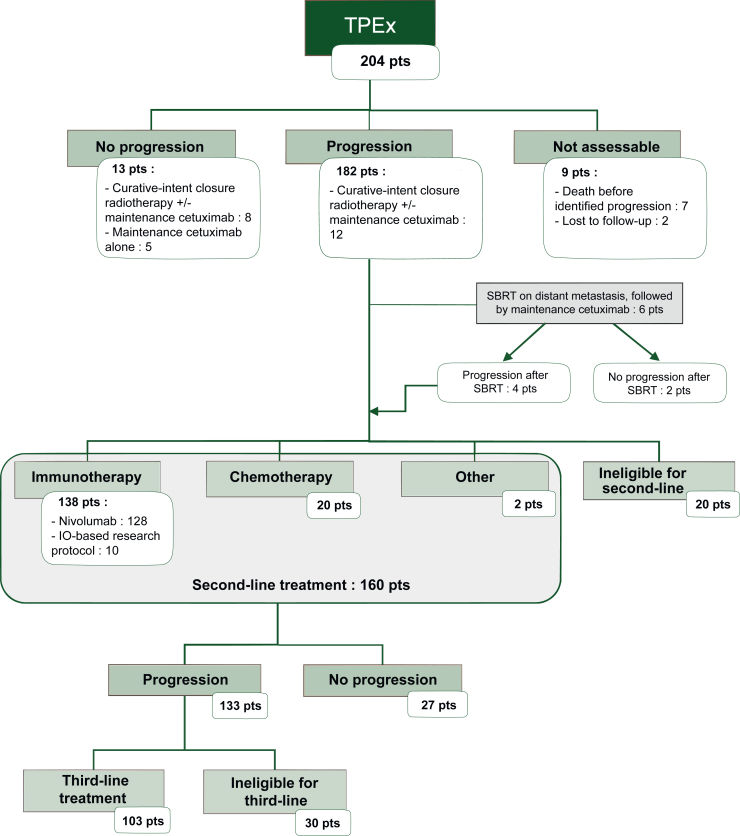
**Follow-up chart of patients’ evolution under TPEx.** IO, immunotherapy; Pts, patients; SBRT, stereotactic body radiation therapy; TPEx, docetaxel, platinum, cetuximab.

After a median follow-up of 35.8 months (IQR 23.7-44.3 months), median PFS1 was 6.0 months (95% CI 5.7-6.9 months) (Figure 1). A total of 182 patients progressed during or after TPEx regimen, 160 of whom (87.9%) received a systemic second-line treatment: 128 patients (70.3%) were treated with nivolumab, 20 (11.0%) with chemotherapy-based treatment and 10 (5.5%) were included in an IO-based research protocol. Patients’ outcomes under TPEx are detailed in Figure 2 and Supplementary Table S1 (available at https://doi.org/10.1016/j.esmoop.2025.104544). At the end of data collection, 11 patients were still on maintenance by cetuximab and 150 were dead. Two patients were lost to follow-up; one of them had received a curative-intent radiotherapy.

Median PFS2 was 2.5 months (95% CI 2.0-2.8 months), and median time from progression on second-line treatment to death was 5.4 months (IQR 2.2-10.3 months). Median PFS2^IO^ was 2.3 months (95% CI 1.9-2.8 months) (Supplementary Figures S1 and S2, available at https://doi.org/10.1016/j.esmoop.2025.104544). A total of 133 patients progressed under second-line treatment; 103 patients were exposed to a systemic third-line treatment.

Median OS in the entire population was 17.9 months (95% CI 15.7-19.6 months) (Figure 1). Overall, among the 182 patients who progressed under TPEx, 148 patients were exposed to IO (81.3%), either as a second-line (75.8%) or third-line treatment or more (5.5%).

### Subgroup analysis among patients with available CPS

Baseline characteristics were not different between patients with or without CPS available (Supplementary Table S2, available at https://doi.org/10.1016/j.esmoop.2025.104544). The availability of CPS increased from 10.5% in patients treated between 2018 and 2019 to 50.6% in patients treated between 2020 and 2023.

The CPS ≥1 population had significantly more clinically threatening diseases compared with the CPS <1 population (48.5% versus 21.8%, *P* = 0.009, Supplementary Table S3, available at https://doi.org/10.1016/j.esmoop.2025.104544). Regarding OS, patients with a CPS <1 had a numerically longer survival than patients with CPS ≥1 (20.9 months versus 15.4 months, respectively, *P* = 0.046, Supplementary Figure S3, available at https://doi.org/10.1016/j.esmoop.2025.104544). CPS status was not associated with PFS1 or PFS2^IO^ (Supplementary Figures S4 and S5, available at https://doi.org/10.1016/j.esmoop.2025.104544). Due to the low number of patients with CPS available, this variable was not assessed in the univariate/multivariate analysis.

### Univariate and multivariate analysis

In univariate analysis of baseline characteristics, having a clinically threatening disease, presence of liver metastases and time to relapse <6 months after localized HNSCC were significantly associated with increased mortality (Table 3). In multivariate analysis, having a clinically threatening disease, use of carboplatin and time to relapse <6 months were significantly associated with increased mortality. Time to relapse <6 months and age <62 years old were significantly associated with PFS1 in multivariate analysis (Supplementary Table S4, available at https://doi.org/10.1016/j.esmoop.2025.104544).

**Table 3:** Univariate and multivariate OS analysis (Cox model)

			Univariate analysis		Multivariate analysis	
	Log rank (*P* value)	*n*	HR (95% CI)	*P*	HR (95% CI)	*P*
Sex: male (ref: female)	0.07	204	1.58 (0.96-2.58)	0.07		
Age: ≥62 years old (ref: <62)	0.2		0.81 (0.58-1.11)	0.2		
Clinically threatening disease (ref: no)	0.003		1.65 (1.18-2.30)	0.004	1.69 (1.18-2.40)	0.004
Liver metastases (ref: no)	0.04		1.81 (1.02-3.21)	0.04	1.47 (0.80-2.70)	0.20
Carboplatin (ref: cisplatin)	0.05		1.38 (1-1.91)	0.05	1.57 (1.11-2.21)	0.01
History of localized HNSCC: no (*de novo* metastatic) [ref: yes (recurrent), time to relapse >6 months^a^]	0.08		1.06 (0.7-1.6)	0.8	1.03 (0.67-1.60)	0.9
History of localized HNSCC: yes (recurrent), time to relapse ≤6 months [ref: yes (recurrent), time to relapse >6 months^a^]	0.08		1.54 (1.04-2.27)	0.03	1.54 (1.03-2.30)	0.04

CI, confidence interval; HNSCC, head and neck squamous cell carcinoma; HR, hazard ratio; OS, overall survival; ref, reference; R/M, recurrent and/or metastatic.

a Between last treatment of localized HNSCC and R/M HNSCC diagnosis.

## Discussion

In this multicentric retrospective study, patients treated with TPEx in first-line setting for an R/M HNSSC had a median OS of 17.9 months. Among patients who progressed under TPEx, 81.3% were exposed to IO in subsequent lines and 56.6% received at least three lines of treatment.

The 17.9 months OS in this study compares favorably with data from prospective studies based on a chemotherapy-cetuximab regimen, in which median OS varies from 10.3 to 14.6 months.^9^^,^^12^^,^^11^^,^^16^ Of note, the patients evaluated in the present study were selected based on their PS status (0 or 1), but this is also the case in phase III clinical trials. Age, sex and proportion of metastatic disease, however, were similar between this study and TPExtreme^11^ or Keynote048.^9^ PFS1 (6 months) is highly comparable with the data observed in the TPExtreme study^11^ (6 months) as well as the proportion of patients with cetuximab maintenance initiation (75% versus 72%). In the same line, PFS2^IO^ (2.3 months) is comparable with the PFS observed in Checkmate 141 (2.0 months),^13^ evaluating nivolumab in second line after progression on platinum-based chemotherapy.

In the total population, the very high rate of patients who received second-line treatment (78%) and were exposed to IO (72.5%) in this study contrasts with rates observed in studies based on a chemotherapy-cetuximab-based regimen, in which subsequent IO exposure rate varied from 16.5% to 46%.^9^^,^^12^^,^^11^^,^^16^ This difference may be explained by the fact that we included patients from 2018, when nivolumab became available in France, while many of the large phase III studies started inclusions before that date. Moreover, all patients were included in French centers, where nivolumab is reimbursed and thus easily available.

The potential interest of a sequence starting with a chemotherapy-cetuximab-based regimen followed by IO at progression has already been suggested. An exploratory analysis of TPExtreme^11^ showed a median OS of 21.9 months for patients in the TPEx arm receiving second-line IO, a result comparable to that of KESTREL^16^ in case of EXTREME followed by IO (22.4 months). These data should be interpreted with caution since there is a bias of selection (exclusion of patients with early progression or not amenable to second line). The results of the present study suggest that this kind of sequence may be of interest, in particular in case of low/unknown CPS or contraindication to 5-fluorouracil.

Some 62.5% of patients had a CPS <1, which is higher than other similar populations (43% in Checkmate141^13^ and 15% in Keynote048^9^), but consistent with the fact that TPEx is mostly used in PD-L1-negative tumors. For patients with CPS available, no correlation between CPS and PFS1 or PFS2^IO^ was found. This can be related to the high rate of patients exposed to IO in our population and the known efficacy of nivolumab in the second-line setting regardless of PD-L1 status.^13^^,^^17^ Regarding the correlation between CPS and OS, we observed a marginally significantly better OS for patients with CPS <1. This result should be interpretated with caution, however, since it could be biased by the higher rate of clinically threatening disease in the CPS ≥1 population. Among the 88 patients with available CPS, 5 had their tumor tissue sampled for CPS analysis after the initiation of TPEx, potentially affecting the interpretation of survival outcomes, as treatment decisions were made without prior knowledge of CPS status. Of note, due to the low number of patients with CPS available, we did not carry out a multivariate analysis of prognostic markers.

This study has some limitations. First, due to its retrospective design, some data were missing, especially concerning CPS status, which was available for only 43.1% of the population. The lack of CPS data was particularly notable in 2018-2019, likely due to the publication of Keynote048^9^ in late 2019 and the fact that this study was conducted in French centers, where the HAS (French National Authority for Health) only approved pembrolizumab reimbursement for head and neck cancer in June 2020. Second, CPS were analyzed locally and not in a centralized laboratory. Third, we do not have the reasons for selection of the TPEx regimen instead of IO or chemo-IO for patients with CPS ≥1. Of note, very few patients (8/88, 9%) had a CPS ≥20 in this study, compared with 43% in Keynote048. Moreover, 48.5% of patients had clinically threatening diseases among the CPS ≥1 population. Knowing that TPEx has a high expected objective response rate (59%),^11^ we can assume that this regimen was chosen over an IO-based treatment of CPS ≥1 tumors with clinically threatening features, leading to a higher rate of clinically threatening disease and hence to a potentially biased poorer OS in our CPS ≥1 population. Of note, no large-scale prospective study has compared TPEx and IO-chemotherapy in patients with clinically threatening disease. Fourth, this study was conducted in French cancer centers experienced in the field of HNSCC. This may have led to a more intensive treatment with a potential impact on OS. Indeed, half of patients (103/204), were exposed to at least three lines in the R/M setting, and some patients had a curative-intent closure radiotherapy or stereotactic body radiation therapy on distant oligometastases, which improves survival in that setting.^18^, ^19^, ^20^ Finally, the toxicity profile was not collected due to the retrospective nature of the study and the risk of partial or incomplete reports in patients’ medical files.

## Conclusion

In conclusion, although retrospective, this large multicentric study reports a real-life 17.9 months OS in R/M HNSCC treated by TPEx as a first-line treatment, that compares favorably with historical data. A very high rate (>80%) of patients who progressed under TPEx were exposed to IO after progression. These results provide reassuring data regarding the use of TPEx as a first-line treatment for patients with low/unknown CPS or contraindication to the 5-fluorouracil–platinum–pembrolizumab regimen.
