# Supplementary material for: Retrospective multicentric survival analysis of patients receiving TPEx regimen as first-line treatment of recurrent and/or metastatic head and neck squamous cell carcinoma
Source: ESMO Open. 2025 Apr 11;10(4):104544. doi: 10.1016/j.esmoop.2025.104544 (PMC12017985; doi:10.1016/j.esmoop.2025.104544)
Supplement: Figure S1 [file mmc1.docx]

**Figure S1. Kaplan-Meier PFS2 analysis in total population**


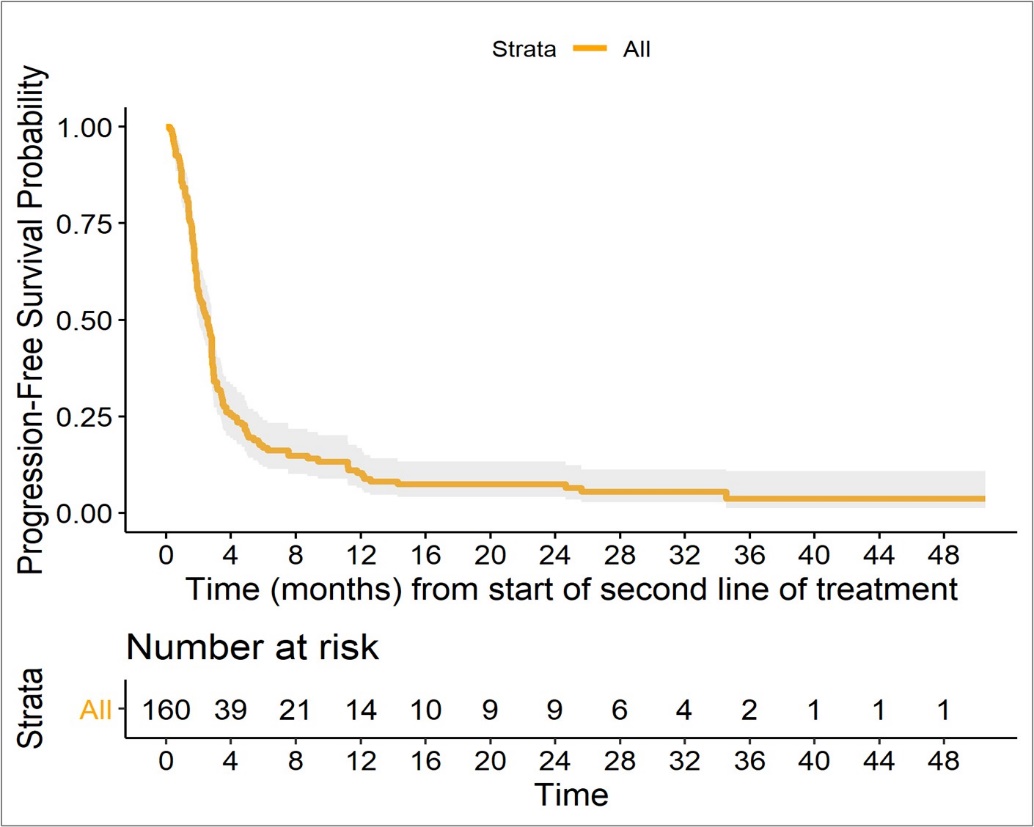


**Median PFS2** : 2.5 months (95% CI 2.0-2.8)

PFS2=Progression-free survival 2; CI=confidence interval.
