# Supplementary material for: Retrospective multicentric survival analysis of patients receiving TPEx regimen as first-line treatment of recurrent and/or metastatic head and neck squamous cell carcinoma
Source: ESMO Open. 2025 Apr 11;10(4):104544. doi: 10.1016/j.esmoop.2025.104544 (PMC12017985; doi:10.1016/j.esmoop.2025.104544)
Supplement: Figure S2 [file mmc2.docx]

**Figure S2. Kaplan-Meier PFS2 analysis according to 2^nd^ line treatment**


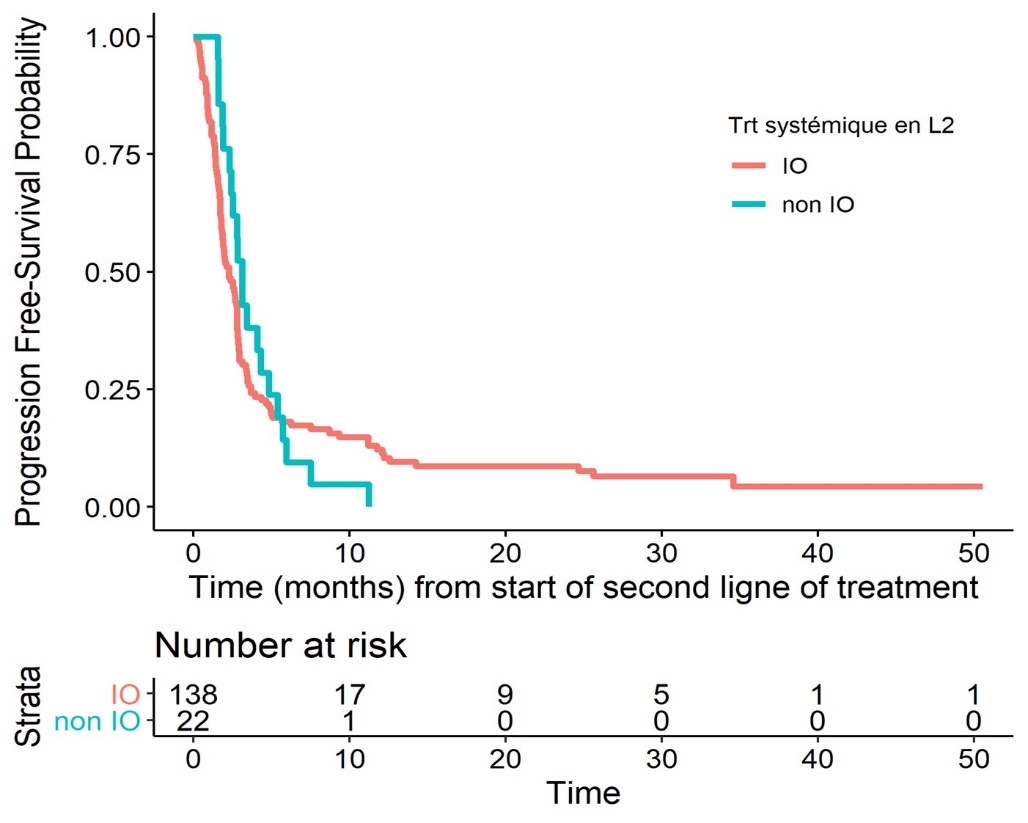


p = 0.65

PFS2=Progression-free survival 2; PFS2^io^=Progression-free survival 2 under immunotherapy; PFS2^non-io^=Progression-free survival 2 under treatment other than immunotherapy; IO=immunotherapy.

line
