# Supplementary material for: Retrospective multicentric survival analysis of patients receiving TPEx regimen as first-line treatment of recurrent and/or metastatic head and neck squamous cell carcinoma
Source: ESMO Open. 2025 Apr 11;10(4):104544. doi: 10.1016/j.esmoop.2025.104544 (PMC12017985; doi:10.1016/j.esmoop.2025.104544)
Supplement: Figure S5 [file mmc5.docx]

**Figure S5. Kaplan-Meier PFS2^IO^ analysis according to CPS**


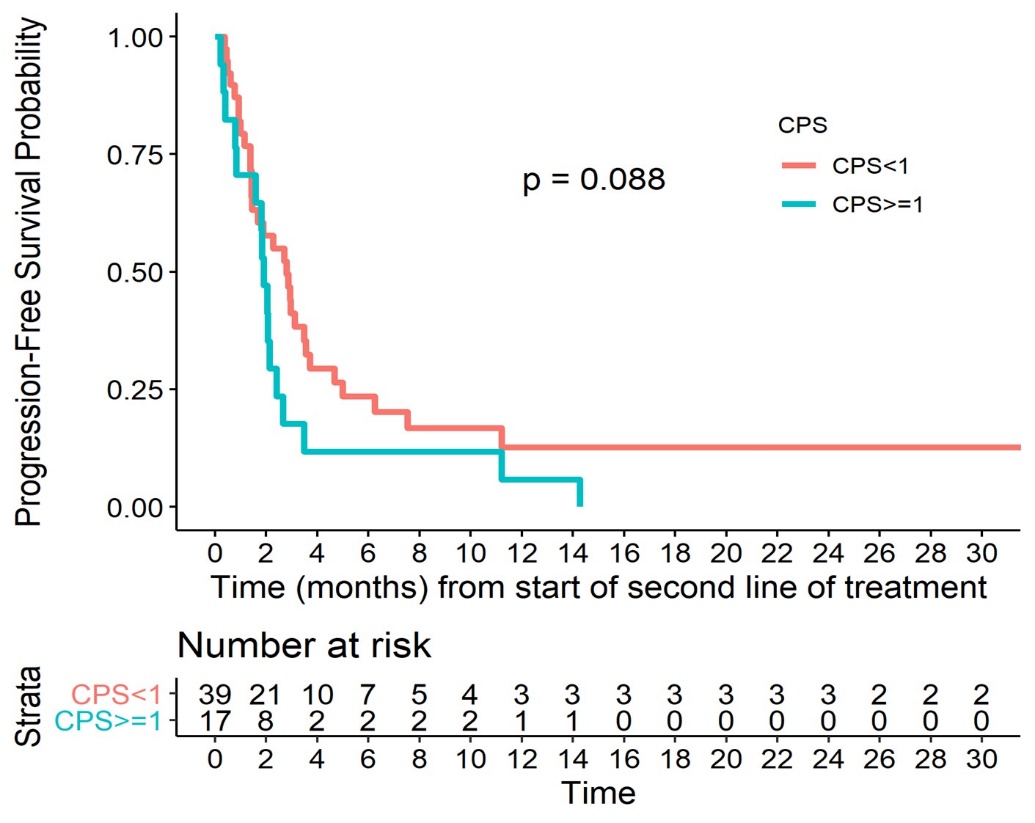


PFS2^io^=Progression-free survival 2 under immunotherapy; CPS=combined positive score.
