# Supplementary material for: Retrospective multicentric survival analysis of patients receiving TPEx regimen as first-line treatment of recurrent and/or metastatic head and neck squamous cell carcinoma
Source: ESMO Open. 2025 Apr 11;10(4):104544. doi: 10.1016/j.esmoop.2025.104544 (PMC12017985; doi:10.1016/j.esmoop.2025.104544)
Supplement: Supplementary Appendix [file mmc6.docx]

**Supplementary Appendix**

Supplementary data related to this article can be found at Supplementary Appendix, available at <https://doi.org/10.1016/j.esmoop.2025.104544>.
